# Effects of icotinib with and without radiation therapy on patients with EGFR mutant non-small cell lung cancer and brain metastases

**DOI:** 10.1038/srep45193

**Published:** 2017-03-23

**Authors:** Yun Fan, Yanjun Xu, Lei Gong, luo Fang, Hongyang Lu, Jing Qin, Na Han, Fajun Xie, Guoqin Qiu, Zhiyu Huang

**Affiliations:** 1 Wenzhou Medical University, Wenzhou, China; 2 Department of Thoracic Oncology, Zhejiang Cancer Hospital, Hangzhou, China; 3 Key Laboratory Diagnosis and Treatment Technology on Thoracic Oncology and Cancer Research Institute, Hangzhou, China; 4 Department of Pharmacy, Zhejiang Cancer Hospital, Hangzhou, China; 5 Department of Radiation Oncology, Zhejiang Cancer Hospital, Hangzhou, China

## Abstract

EGFR-TKIs and radiation therapy (RT) are the principal treatment for patients with brain metastases (BM) and EGFR mutant NSCLC. However, the optimal use of brain RT for patients with asymptomatic BM remains undefined. A total of 152 patients were identified. 58 patients were excluded. Of the remaining 97 patients, 56 patients received upfront RT followed by icotinib, including WBRT or SRS. 41 patients received icotinib therapy alone. The mOS from diagnosis of BM was 27.0 months for the whole cohort (95% CI, 23.9–30.1 months). There was no difference in OS between the RT followed by icotinib group and the icotinib alone group (31.9 *vs.* 27.9 months, *P* = 0.237), and similar results were found in the SRS subgroup (35.5 vs. 27.9 months, *P* = 0.12). Patients with the *EGFR* Del19 mutation had a longer OS than patients with the exon 21 L858R mutation (32.7 vs. 27.4, *P* = 0.037). Intracranial progression-free survival (PFS) was improved in the patients who received RT followed by icotinib compared to those receiving icotinib alone (22.4 vs. 13.9 months, *P* = 0.043). Patients with EGFR-mutant adenocarcinoma and BM treated with icotinib exhibited prolonged survival. A longer duration of intracranial control was observed with brain RT.

Over the last several decades, lung cancer has become the leading cause of cancer-related mortality worldwide for both men and women^1^. Non-small cell lung cancer (NSCLC) comprises 80% to 85% of lung cancers. NSCLC adenocarcinomas are characterized by a high incidence of central nervous system (CNS) metastasis. Approximately 30% of patients with NSCLC will develop brain metastases (BM) during the course of their illness^2^. The incidence rate of BM is higher among patients with epidermal growth factor receptor (*EGFR*) mutations than among patients with wild-type *EGFR*^3^,^4^. Historically, whole brain radiation therapy (WBRT) and stereotactic radiosurgery (SRS), either alone or in combination, have been the standard treatments for BM. However, the presence of BM in patients with NSCLC leads to a poor prognosis with a median survival of only 7 months after traditional standard care^5^.

Over the last decade, EGFR tyrosine kinase inhibitors (TKIs) have been recognized as the standard first-line therapy for patients with activating *EGFR* mutations. Several randomized studies supporting this paradigm shift have demonstrated that treatment with TKIs results in higher tumor response rates and longer progression-free survival (PFS) compared to treatment with cytotoxic chemotherapy^6^,^7^,^8^. Phase II trials and retrospective studies have also demonstrated that treating BM in patients with *EGFR*-mutant NSCLC with EGFR-TKIs without the use of upfront radiation produces better efficacy than treatment including upfront radiation^9^,^10^. More recent data examining survival in a population of patients with BM and *EGFR* mutations have shown a median overall survival of 21.9–26 months from the time of BM incidence^11^,^12^.

Reversible EGFR-TKIs, such as gefitinib and erlotinib, have proven effective for treating BM in patients with *EGFR*-mutant NSCLC. Icotinib, another first-generation EGFR-TKI, produces similar PFS to gefitinib in patients with advanced NSCLC^13^. Moreover, there is also evidence of the effectiveness of icotinib in *EGFR*-mutant patients with BM^14^. Current treatment algorithms recommend surgery or SRS for oligometastatic and symptomatic BM^15^,^16^,^17^. For patients with multiple BM, WBRT remains the standard of care^18^. However, WBRT is associated with potential long-term neurocognitive toxicities, which are concerning when considering prolonging the life of patients with BM and *EGFR* mutations. The relative benefits of upfront EGFR-TKIs combined with radiation therapy (RT) versus EGFR-TKIs alone for *EGFR*-mutant patients with BM have not been determined. Although one retrospective analysis suggested that the use of upfront EGFR-TKI and the deferral of SRS or WBRT may result in inferior overall survival (OS) for such patients^19^, no randomized study has been conducted to compare the effects of upfront EGFR-TKI with RT and those of upfront RT combined with EGFR-TKI on disease progression. Thus, the optimal treatment strategy for BM with *EGFR* mutations remains controversial. We therefore conducted the current retrospective study to compare upfront RT combined with icotinib to icotinib alone as first-line therapies for patients with *EGFR*-mutant NSCLC and BM as well as to identify prognostic factors that could be used to tailor treatment strategies.

## Results

### Patient characteristics

A total of 152 patients with metastatic *EGFR*-mutant adenocarcinoma with BM who were diagnosed and received icotinib therapy between October 2011 and October 2014 were identified. Of these, 55 patients were excluded from analysis: 34 had developed BM while receiving icotinib, 10 patients had *EGFR* mutations outside of the exon 19 deletion and exon 21 858R mutations, 8 patients lacked complete clinical and imaging data, and 3 patients were lost to follow-up. The remaining 97 patients were enrolled in the study. All of the enrolled patients had histologically proven NSCLC with radiographically confirmed BM, and all received icotinib therapy. Fifty-six patients were treated with upfront RT combined with icotinib, and 41 patients received icotinib therapy alone. The patient characteristics at baseline are detailed in Table 1. The median age was 58 years (range, 31 to 77 years). The majority of patients were women (56%), had never been smokers (68%), had a KPS of 70–90 (87%), presented with extracranial disease (73%), had asymptomatic BM (73%), had diagnosis-specific GPA scores of 0.0–2.0 (74%), had exon 19 deletions (59%) and had received upfront RT combined with icotinib therapy (58%). About half of the patients had four or more BM (n = 47, 49%). The patients receiving RT combined with icotinib were more likely to be symptomatic from their BM (37% vs. 12%, *P* = 0.005) and had a greater number of BM (59% vs. 34%, *P* = 0.054). In the icotinib group, there was a higher percentage of extracranial disease (90% vs. 61%, *P* = 0.003).

There were no significant differences in the *EGFR* mutation subtypes or the disease-specific GPA scores between the groups, and no other significant differences were found.

### Treatment characteristics

A total of 56 of the 97 patients received RT for treatment of brain metastases, while 41 of the patients received TKI therapy alone. Surgical resection preceded RT in nine patients. RT was delivered with either localized SRS (Gamma Knife) or WBRT. Forty-six patients received WBRT, with a median dose of 3000 cGy in 10 fractions (range, 3000–3900 cGy). Of the 10 patients treated with SRS, 9 (90.0%) were treated with a single fraction to 1 lesion (60.0%) or multiple lesions (40.0%). The median dose of single-fraction SRS was 2000 cGy (range, 1500–2500 cGy). Twenty-one patients had first line chemotherapy before study enrollment, with the most common regimens being platinum combined with gemcitabine, docetaxel or pemetrexed. Of the patients treated with icotinib alone, 48.8% (n = 20) were seen by a radiation oncologist before the decision to treat with icotinib. Twenty-seven patients (65.9%) never received brain radiation. The patients who eventually received RT (n = 14) did so at a median of 12 months (range, 8–26 months) after the diagnosis of BM.

### Outcomes

**Figure 1:**
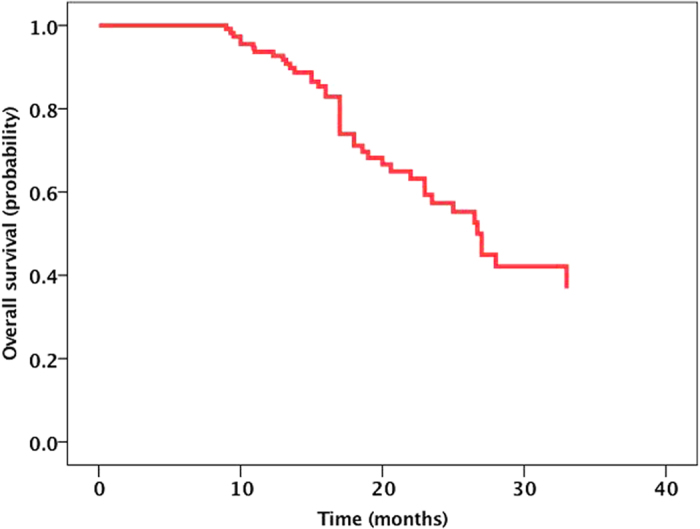
The Kaplan-Meier curve for OS. The median OS from diagnosis of BM was 27.00 months (95% CI, 23.9 to 30.1 months).

**Figure 2:**
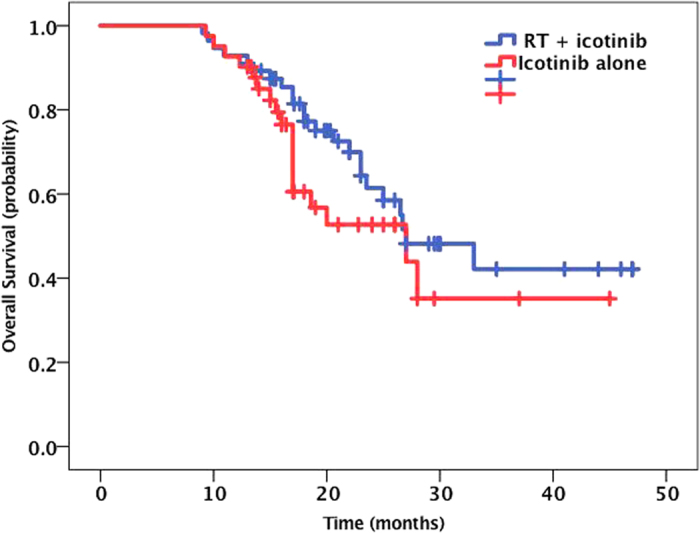
The Kaplan-Meier curve for OS between RT + icotinib group and icotinib alone group. The patients in the RT combined with icotinib group showed a longer OS (median time, 31.9 months) than the patients in the icotinib alone group (median time, 27.9 months), but with no statistically significance (P = 0.237).

**Figure 3:**
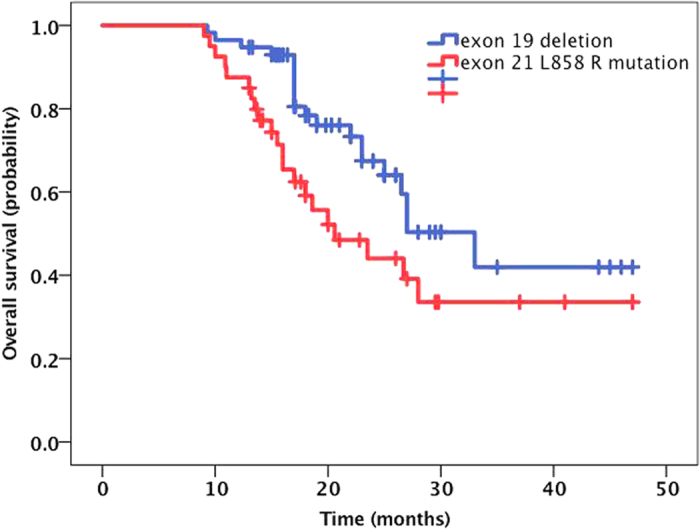
The Kaplan-Meier curve for OS between different mutant groups. The median OS was 32.7 months (95% CI 28.1–37.3) in the EGFR del19 mutation group and 27.4 months (95% CI 22.1–32.7) in the exon 21 L858R group (P = 0.037).

**Figure 4:**
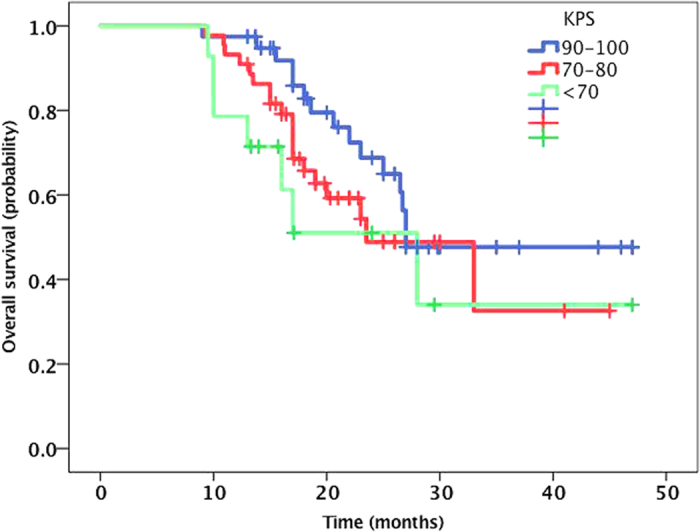
The Kaplan-Meier curve for OS between different KPS groups. There was no survival difference for patients based on KPS, with a median survival of 33.6 months for a KPS of 90 to 100, 28.7 months for a KPS of 70 to 80, and 27.1 months for a KPS of 70 (P = 0.22).

Fifty-six patients were alive at the time of this analysis, and the median follow-up time was 28.5 months (range, 19.6 to 48 months). The median OS from diagnosis of BM was 27.00 months (95% CI, 23.9 to 30.1 months). The Kaplan-Meier curve for OS is shown in Figure 1. The 1-year, 2-year and 3-year OS rates were 93.1%, 58.7%, and 42.4%, respectively. The patients in the RT combined with icotinib group showed a longer OS (median time, 31.9 months) than the patients in the icotinib alone group (median time, 27.9 months), but the difference was not statistically significant (*P* = 0.237) (Fig. 2). The median OS was also similar between the SRS plus icotinib group and the icotinib alone group (35.5 vs. 27.9 months, *P* = 0.12). In subgroup analyses examining different *EGFR* mutation types, we noted a statistically significant improvement in median OS for the patients with the *EGFR* del19 mutation compared to those with the exon 21 L858R. The median OS was 32.7 months (95% CI 28.1–37.3) in the *EGFR* del19 mutation group and 27.4 months (95% CI 22.1–32.7) in the exon 21 L858R group (*P* = 0.037) (Fig. 3). There was no survival difference for patients based on KPS, with a median survival of 33.6 months for a KPS of 90 to 100, 28.7 months for a KPS of 70 to 80, and 27.1 months for a KPS of 70 (*P* = 0.22; Fig. 4). There was also no difference between a KPS of 90 to 100 and a KPS <90. In the UVA, only the exon 19 mutation was a predictor for improved OS. In the MVA, the exon 19 mutation was found to predict OS (*P* = 0.096). Notably, gender, age, smoking history, KPS, number of metastatic lesions in the brain (one *vs.* multiple), absence of extracranial disease, type of treatment (have/no RT) and treatment regimens of icotinib were not observed to significantly influence prognosis.

**Figure 5:**
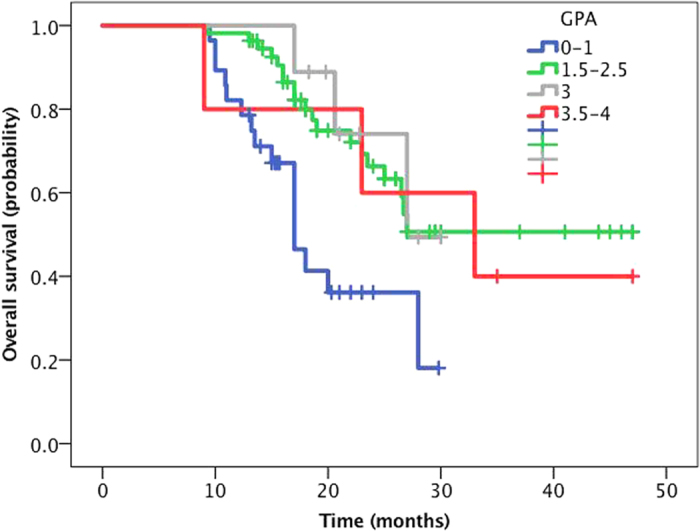
The Kaplan-Meier curve for OS between different GPA groups. The patients were divided into NSCLC-specific GPA groups, and the median survival was calculated for each of these groups. The OS of the group with a GPA score of 0–1 was significantly worse than those for the others (19.8 months, P = 0.006).

To assess the utility of the GPA to predict survival for the patients with *EGFR*-mutant NSCLC and BM, the patients were divided into NSCLC-specific GPA groups, and the median survival was calculated for each of these groups. The OS of the group with a GPA score of 0–1 was significantly worse than those for the others (19.8 months, *P* = 0.006, Fig. 5). This result suggested that the GPA could predict survival for this patient population.

**Figure 6:**
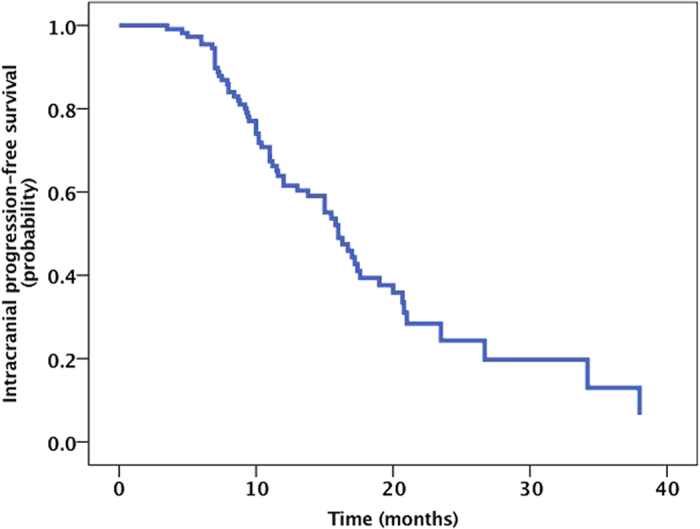
The Kaplan-Meier curve for IC-PFS. The median IC lesion PFS for the entire cohort was 16.0 months (95% CI, 13.5 to 18.6 months).

**Figure 7:**
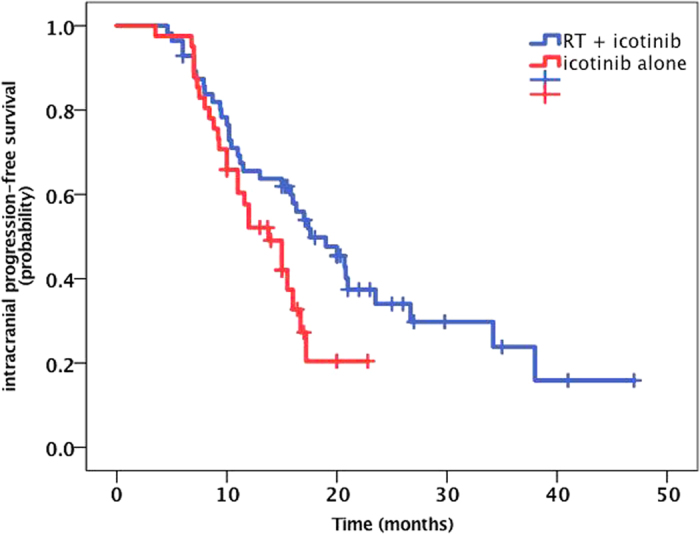
The Kaplan-Meier curve for IC-PFS between RT + icotinib group and icotinib alone group. The IC PFS in the RT plus icotinib group was greater than that for the patients treated with icotinib alone (22.4 vs. 13.9 months, P = 0.043).

**Figure 8:**
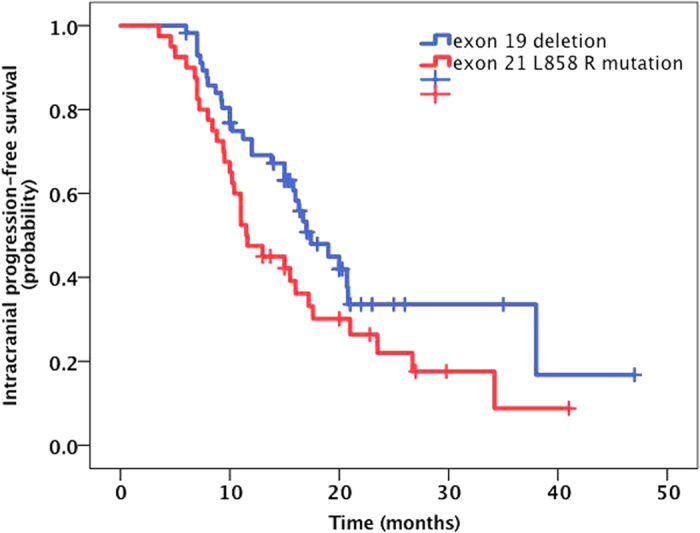
The Kaplan-Meier curve for IC-PFS between different mutant groups. The median IC PFS for the EGFR del19 mutation group was greater than that for the exon 21 L858R mutation group (23.3 vs. 16.8 months, P = 0.06).

The median IC lesion PFS for the entire cohort was 16.0 months (95% CI, 13.5 to 18.6 months) (Fig. 6). The 1-year, 2-year and 3-year IC PFS rates were 62.3%, 22.4%, and 11.5%. The IC PFS in the RT plus icotinib group was greater than that for the patients treated with icotinib alone (22.4 vs. 13.9 months) (Fig. 7), and this finding was statistically significant (*P* = 0.043). However, the IC PFS for the SRS group was not longer than that for the group receiving icotinib alone (17.8 vs. 13.9 months, *P* = 0.56). The median IC PFS for the *EGFR* del19 mutation group was also greater than that for the exon 21 L858R mutation group (23.3 vs. 16.8 months) (Fig. 8), although the difference was not statistically significant (*P* = 0.06). In the MVA, none of the assessed factors retained significance.

IC lesion response rates (according to RECIST criteria) were evaluated between 2 and 4 months after initiation of therapy. The results showed that 32.6% of the patients had a complete response (CR, n = 28), 50.0% had a partial response (PR, n = 43), 2.3% had progressive disease (PD, n = 2), and 15.1% had stable disease (SD, n = 13), for an overall response rate (ORR) of 82.6% among those assessed for response (n = 86). The ORR was slightly higher in the RT combined with icotinib group, but the difference was not statistically significant (86.0% vs. 77.8%; *P* = 0.10). Extra-cranial lesions response rates were also evaluated. The results showed that 8.2% of the patients had a CR (n = 7), 60.0% of the patients had a PR (n = 51), 23.5% of the patients had a SD (n = 20), 8.2% of the patients had a PD (n = 7), and the ORR was 68.2%. Median PFS of extra-cranial lesions is 11.4 months (95% CI: 7.1–20.5).

Overall, the most prevalent site of first failure was extra-axial in 45 patients (46.4%), the CNS in 17 patients (17.5%), and both the CNS and systemically in 8 patients (8.2%). For 27 patients (27.8%), there was no failure, either systemically or in the brain. Only 10.9% of the patients treated with WBRT experienced failure in the brain as a component of first failure compared with 41.4% of the patients treated with icotinib alone and 30.0% of the patients treated with SRS. And, these differences were statistically significant (*P* = 0.02).

## Discussion

In the current study, for the entire cohort of patients, the median survival was 27 months, the median IC PFS was 16 months, and the remission rate was 82.6%. This result is similar to previous reports of the efficacy of erlotinib and gefitinib in the treatment of *EGFR*-mutant patients with BM in phase II clinical trials^9^,^10^,^20^. Icotinib can pass through the blood brain barrier to treat brain lesions. Although similar to other first generation EGFR-TKIs^21^,^22^, the permeable rate for icotinib passing through the blood brain barrier is low, at approximately 1.4%^14^.

Although for the baseline clinical characteristics, the patients in the icotinib combined with local RT group had more symptoms of BM and more frequently exhibited a brain metastasis foci number of greater than or equal to four, the subgroup analysis showed that the combined treatment group had a longer IC PFS compared to the group treated with icotinib alone (22.4 vs. 13.9 months, P = 0.043). This result demonstrates that brain RT has great significance for the control of brain lesions and related symptoms. This is consistent with results reported by other researchers. However, in the present study, there was no significant difference in the IC PFC between the patients in the gamma knife treatment subgroup and the patients in the icotinib alone group (17.8 vs. 13.9 months, P = 0.56). Moreover, the brain lesion recurrence rate in the gamma knife subgroup was higher than that in the WBRT group (30% vs. 10.9%), which is consistent with the results reported by Gerber *et al*., who found a significantly higher failure rate for brain lesions in a gamma knife group compared to a WBRT group (71% vs. 24%, P = 0.004)^12^. Other studies have also reported no significant differences in OS in patients with 1–3 brain metastasis lesions treated with SRS vs. SRS+WBRT. In two previous studies, the recurrence rate of cranial cerebral lesions was higher following treatment with SRS alone; however, the cognitive impairment experienced by these patients was significantly reduced, and they had a better quality of life^22^,^23^. Therefore, when choosing local RT for patients with BM, the advantages of SRS may be most pronounced regarding the protection of cognitive function for these patients.

Our OS analysis showed no significant differences between the RT combined with icotinib group and the icotinib alone group (31.9 vs. 27.9 months, P = 0.23), which was consistent with the findings of QUARTZ study. The QUARTZ study showed no survival benefit to addition of WBRT +/− palliative systemic therapy vs. supportive measures only +/− supportive therapy^24^. The OS of the gamma knife treatment group was also not significantly improved compared to that of the icotinib alone group (35.4 vs. 27.9, P = 0.12). This result differs from the studies reported by Gerber^12^ and William (http://www.ClinicalTrials.gov (NCT01724801)). In both of the referenced studies, the OS following SRS treatment was significantly better than that following treatment with an EGFR-TKI alone (64 vs. 26 months, P = 0.004; 58.4 vs. 19.1 months, P < 0.001; respectively). Additionally, the OS following WBRT was also improved compared to that following treatment with EGFR-TKI alone, although the difference was not significant (35 vs. 26 months, P = 0.62; 29.9 vs. 19.1 months, P = 0.09).

The differences in the results between our study and the other referenced studies may be related to the following factors. First, in our study, there were no significant differences in the GPA scores among the different groups. However, the patients in the icotinib alone group did have a higher rate of IC metastases, and more patients with cerebral symptoms were found in the combined therapy group. Second, with regard to therapeutic effect, 17 of the patients in the icotinib alone group (41 cases) underwent WBRT or SRS after already experiencing failure with icotinib therapy. In other words, 41.5% of the patients in the icotinib alone group still accepted brain RT after brain metastasis, which may have produced similar OS values for the two groups of patients. This confirms the good therapeutic effect of the combination of EGFR-TKI and cranial RT for *EGFR*-mutant patients with BM. Thus, whether the timing of RT influences prognosis remains to be determined. Third, Gerber’s study reported^12^ that 71% of patients undergoing SRS showed brain lesion recurrence, which was significantly higher than that observed for patients undergoing WBRT (24%). Obviously, for controlling brain lesions, SRS did not outcompete WBRT. Therefore, it is uncertain whether the survival advantage of the SRS treatment group may be derived from the clinical features of the patients themselves due to their reduced number of IC lesions. Considering that the studies reported by Gerber and William and the present study all used retrospective analysis, the total number of cases is small (110 cases, 50 cases and 97 cases, respectively), and there were few patients in the SRS treatment groups (15 cases, 17 cases and 10 cases, respectively). Thus, the optimal timing for local RT and the survival advantage imparted by SRS both remain to be determined. In a prospective phase II study conducted in Japan that treated *EGFR*-mutant patients with BM with gefitinib as a first-line therapy followed by WBRT as a salvage therapy, the IC PFS following gefitinib treatment was 14.5 months, the OS was 21.9 months, and the patients showed not only longer survival but also a reduced incidence of cognitive impairment.

Therefore, the strategy of first providing TKI-EGFR alone to patients with asymptomatic BM followed by local treatment as salvage therapy when IC lesions are identified may be a good therapeutic option. Patients can exhibit different types of BM (i.e., not all BM have similar characteristics to the original lung cancer), and therefore treatments should be applied to address the conditions that are present. In addition to ensuring a curative effect and helping provide a good quality of life to the patient, the benefits and toxicities of different therapeutic strategies must be considered. Therefore, the optimal timing for brain RT and the parameters used for this should be further explored. There are currently two ongoing multicenter randomized controlled phase II and III studies of *EGFR*-mutant patients with BM, and the results of these studies should provide additional evidence for optimizing the above. Both of these studies are being conducted in China. One trial is randomly comparing the efficacies of WBRT plus erlotinib and erlotinib alone plus WBRT salvage therapy in patients with asymptomatic BM (TRACTS trial, clinicaltrials.gov/NCT01763385). The other trial is randomly comparing the therapeutic effects of first-line icotinib treatment and WBRT in patients with BM greater than or equal to three IC lesions. The enrollment for this latter trial has already been completed (clinicaltrials.gov/NCT01516983).

In our analysis of different EGFR mutation subgroups, we found that patients with the exon 19 deletion mutation had a better OS (27.232.7 vs. 27.4 months, P = 0.037) and a longer IC PFS (23.3 vs. 16.8 months, P = 0.06) than those with the 858 R mutation in exon 21. A number of studies have reported that the efficacies of EGFR-TKIs are distinct among different mutant subtypes. In a small sample of *EGFR*-mutant patients with BM, a Japanese group found that a therapeutic difference still existed. In the present study, we also found that patients with BM and exon 19 deletion mutations in EGFR exhibited better outcomes than patients with the 858R mutation in exon 21, regardless of OS or IC PFS. For patients with the 858R mutation in exon 21, the best means of improving OS, i.e., EGFR-TKIs combined with chemotherapy, anti-angiogenesis therapy, or early intervention with local RT, requires further investigation.

The MVA in the present study showed that age, sex, smoking status, KPS score, cranial metastasis foci, other clinical factors, different treatment plans and different mutation subtypes were all unable to predict the OS for patients in the Cox regression model. Some studies have reported that a KPS score >90 is a good prognostic factor for OS^25^. Our analysis showed that OS was not significantly improved for patients with KPS scores of 90–100 compared to those with scores under 90 (P = 0.12)^9^,^10^,^11^,^12^. With regard to prognosis, the current research is inconsistent with previous results. There are two possibilities for this discrepancy. First, the EGFR-TKIs used were effective for both IC and extracranial lesions in *EGFR*-mutant patients, and the existence of extracranial metastases has little impact on prognosis. Second, a previous study reported that, even in *EGFR*-mutant patients with an ECOG PS score of 3–4, gefitinib can still only obtain a 66% response rate, a median PFS of 6.5 months, and an OS of 17.8 months, while 79% of patients undergoing TKI treatment show an improved PS score^26^. Thus, even with poor physical condition, patients can still benefit from treatment with EGFR-TKIs. Regarding whether GPA prognostic models can predict patient survival, the current study shows that the OS for patients with a 0–1 GPA score (19.8 months) was significantly shorter than that for the other groups, and the difference was statistically significant (P = 0.006). However, a difference in OS was not obvious in the other three groups. Apparently, in the era of molecular typing, the prognostic value of a single factor is reduced, and the exact factors affecting the prognosis for these patients need to be further confirmed.

There are many limitations in the current study. First, we used a retrospective design, and due to the variety of exclusion factors, there may have been bias in choosing patients for enrollment. Therefore, the results reported here are not entirely representative of a large sample. Second, the sample size of this study was quite small, which may have affected its statistical power. Third, based on the baseline clinical characteristics of the patients, the treatment groups were not homogeneous. Fourth, the choice for treatment was not random because it was determined by the willingness of both the physicians and the patients, which may have led to deviations between the two treatment groups. Fifth, in the short-term efficacy evaluation, the different follow-up times may have affected the results. Sixth, follow-up data on cognitive impairment and quality of life were lacking, and we were therefore unable to analyze these factors.

Overall, this study shows that treating *EGFR*-mutant patients with BM with icotinib shows a good therapeutic effect. Furthermore, icotinib combined with local RT produces a longer duration of IC control compared to treatment with icotinib. However, there were no significant differences in OS between the two treatment groups. Additionally, determining the optimal time for local RT requires further investigation through a prospective, multicenter, randomized controlled study.

## Methods

### Patients

We retrospectively evaluated *EGFR*-mutant patients with NSCLC and metastatic dissemination to the brain who had been registered in Zhejiang Cancer Hospital from October 2011 to October 2014. All the patients were aged 18–75 years and had a Karnofsky performance status (KPS) score >50 in addition to histologically or cytologically confirmed NSCLC. Because our purpose was to evaluate the efficacy of icotinib in the treatment of EGFR-TKI-naive patients, we excluded all patients who had experienced BM while already receiving EGFR-TKIs or who did not receive icotinib after SRS or WBRT. For similar reasons, we also excluded patients with *EGFR* mutations other than the exon 19 deletion and 21 858R mutations. Icotinib was given three times daily at a dose of 125 mg each until disease progression or severe toxicity. The research was approved by the ethical committee of the Zhejiang Cancer hospital, and informed consent was obtained from all participants.

Information was collected on baseline variables such as age at diagnosis of BM, sex, stage at diagnosis, smoking history, neurologic symptoms, type of *EGFR* mutation, number and size of BMs on pretreatment imaging, and presence of extracranial metastasis (ECM). Graded Prognostic Assessment (GPA) scores were recorded at the time of brain metastasis diagnoses^27^,^28^. Based on local treatment regimens, the patients were divided into two groups: an RT combined with icotinib group (n = 56) and an icotinib alone group (n = 41). The RT was delivered with either localized SRS (Gamma Knife) or WBRT. Similarly, stratification by *EGFR* mutation type (*EGFR* exon 19 deletions vs. the exon 21 L858R mutation) was applied for the OS and progression-free survival (PFS) analyses. All variables were compared between treatment groups using a global c2 test or Fisher’s exact test for categorical data and one-way analysis of variance for continuous data.

### EGFR mutation analysis

The analysis of *EGFR* mutations was performed by extracting DNA at the hospital. *EGFR* mutations in exons 18–21 were identified by an amplification refractory mutation system (ARMS).

### Efficacy and safety

Assessment of treatment efficacy at the brain level was performed by performing brain magnetic resonance imaging every 2–3 months. Responses rates of tumors in the lung, liver and adrenal gland, if present, were evaluated by computed tomography (CT) scans. Efficacy is reported in terms of objective response rates defined according to the Response Evaluation Criteria in Solid Tumors (RECIST) 1.1^29^ and with regard to PFS and OS. For intracranial (IC) lesions, the PFS was measured from the date of first icotinib intake or first receipt of RT until the date of progression within the brain or the last follow-up. OS was measured from the date of first diagnosis of BM until death or the last survival follow-up.

### Statistical analysis

Patient characteristics are listed by their frequencies for qualitative variables and by the median values and ranges for quantitative variables. OS was calculated from the date of brain metastasis diagnosis to the time of death or cut-off date. IC progression was calculated from the date of beginning icotinib or RT to first progression in the brain. Actuarial progression and survival curves were generated using the Kaplan–Meier method. Univariable (UVA) and multivariable (MVA) analyses were performed using the Cox proportional hazards regression model. A two-sided *P* value < 0.05 was considered statistically significant. Independent predictors of OS were identified by MVA. The log-rank test was used to detect differences between subgroups. Differences among response rates were analyzed by the chi-square test or Fisher’s exact test, as appropriate. Statistical analyses were performed using SPSS for Windows version 13.0 (SPSS, Inc., Chicago, IL, USA).

All methods were performed in accordance with the relevant guidelines and regulations.

## Additional Information

**How to cite this article**: Fan, Y. *et al*. Effects of icotinib with and without radiation therapy on patients with EGFR mutant non-small cell lung cancer and brain metastases. *Sci. Rep.* **7**, 45193; doi: 10.1038/srep45193 (2017).

**Publisher's note:** Springer Nature remains neutral with regard to jurisdictional claims in published maps and institutional affiliations.

## Figures and Tables

**Table 1:** Patient Demographic and Clinical Characteristics.

Factor	Treatment, n(%)			
	Total n = 97(%)	Icotinib+RT n = 56(%)	Icotinib alone n = 41(%)	*P* value
Age at BM diagnosis (year)				
Median age	58	56	59	
<60	55 (57)	36 (64)	19 (46)	
≥60	42 (43)	20 (36)	22 (54)	0.78
Sex				
Male	43 (44)	22 (39)	21 (51)	
Female	54 (56)	34 (61)	20 (49)	0.24
Smoking history				
Never-smoker	66 (68)	40 (71)	26 (63)	
≤10	8 (8)	4 (7)	4 (10)	
>10	23 (24)	12 (22)	11 (27)	0.70
Symptomatic from BM				
No	71 (73)	35 (63)	36 (88)	
Yes	26 (27)	21 (37)	5 (12)	0.005
Brain metastasis at time of diagnosis				
No	21 (22)	13 (23)	8 (20)	
Yes	76 (78)	43 (77)	33 (80)	0.66
NO. of brain metastases at first imaging				
1′	37 (38)	17 (30)	20 (49)	
2–3′	13 (13)	6 (11)	7 (17)	
≥4	47 (49)	33 (59)	14 (34)	0.054
Extracranial metastasis present				
Yes	71 (73)	34 (61)	37 (90)	
No	26 (27)	22 (39)	4 (10)	0.003
Graded prognostic assessment				
0.0–2.0	72 (74)	42 (75)	30 (73)	
2.5–4.0	25 (26)	14 (25)	11 (27)	0.84
EGFR mutation				
Exon 19 deletion	57 (59)	34 (61)	23 (56)	
Exon 21 L858 R	40 (41)	22 (39)	18 (44)	0.65
KPS				
90	40 (41)	24 (43)	16 (39)	
70–80	45 (46)	27 (48)	18 (44)	
<70	12 (13)	5 (9)	7 (17)	0.49
